# Successful Treatment of Phlegmasia Cerulea Dolens by Surgical Thrombectomy: A Case Report

**DOI:** 10.7759/cureus.101127

**Published:** 2026-01-08

**Authors:** Tomofumi Fukuda, Kosuke Saku, Koki Nakamura, Eiji Nakamura, Satoru Tobinaga

**Affiliations:** 1 Cardiovascular Surgery, St. Mary's Hospital, Kurume, JPN

**Keywords:** acute limb ischemia, cyanosis, deep vein thrombosis, phlegmasia cerulea dolens, thrombectomy

## Abstract

Phlegmasia cerulea dolens (PCD) is a rare, life-threatening manifestation of deep vein thrombosis that can clinically resemble acute arterial occlusion. Accurate differentiation is essential for appropriate management. Delayed-phase contrast-enhanced computed tomography is particularly valuable in distinguishing between these two conditions. Here, we report the case of an 86-year-old man who presented with suspected acute limb ischemia, subsequently diagnosed with PCD by contrast-enhanced computed tomography. The patient was successfully treated with emergency surgical venous thrombectomy, followed by postoperative anticoagulant therapy.

## Introduction

Venous thromboembolism, including deep vein thrombosis (DVT), can occasionally result in life-threatening complications [1]. Phlegmasia cerulea dolens (PCD) is a rare and severe manifestation of DVT, characterized by painful limb swelling and cyanosis, which in severe cases can cause limb ischemia [2]. Thus, careful diagnosis is required, as its presentation can mimic acute limb ischemia (ALI) [3], and delayed-phase computed tomography (CT) plays a particularly important role in diagnosing PCD. Here, we present a case of PCD successfully treated with emergency surgical thrombectomy. This case was previously presented at the 122nd Kyushu Regional Meeting of the Japanese Society for Vascular Surgery.

## Case presentation

**Figure 1:**
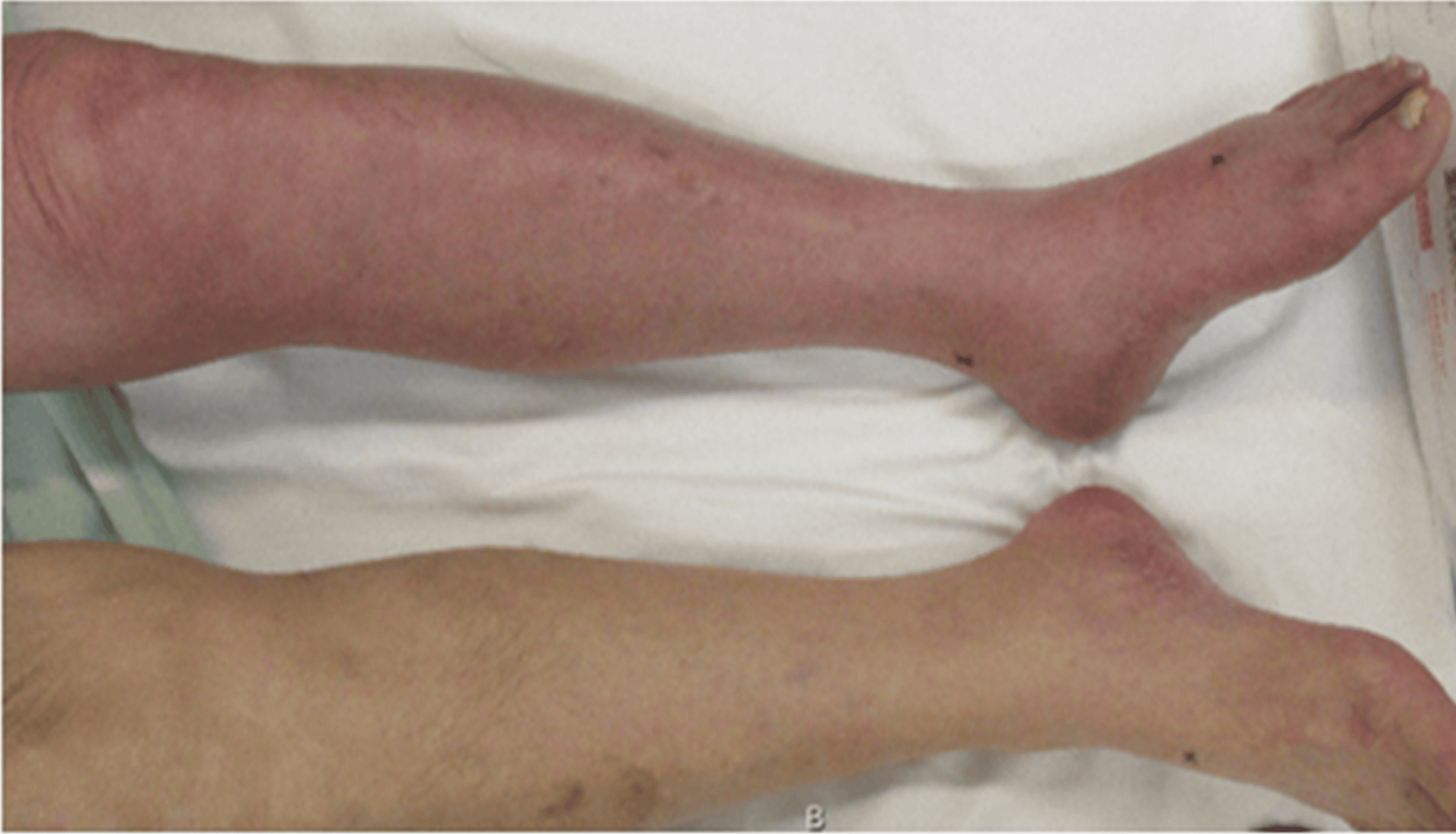
Left lower extremity: swollen, painful, and cyanotic.

**Figure 2:**
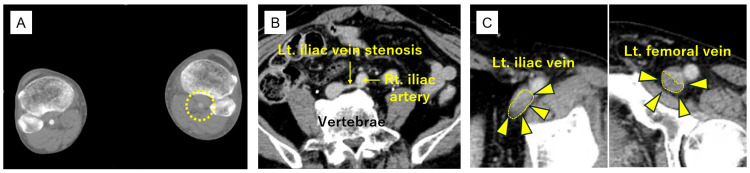
Preoperative computed tomography findings. (A) Contrast-enhanced computed tomography showing delayed opacification in distal arterial segments below the left popliteal artery, without clear occlusion (dashed circle). (B) Left iliac vein is compressed and stenosed between the common iliac artery and the lumbar vertebrae. (C) A filling defect consistent with thrombus (triangle and dashed area) extending from the left iliac vein to the femoral vein. Lt: left; Rt: right.

An 86-year-old man with hypertension presented to our hospital with progressive left leg heaviness and swelling that had developed in the afternoon and subsequently worsened after standing up from a cross-legged sitting position during dinner. On arrival (approximately three hours after this sudden exacerbation), physical examination revealed marked swelling, cyanosis, and pronounced coolness, as well as severe pain in the left lower limb (Figure 1). The degree of muscle tenseness was not sufficient to suggest compartment syndrome. Peripheral arterial pulses distal to the popliteal artery were not palpable, and progressive worsening of paralysis was observed in the left lower limb. Laboratory testing showed elevated D-dimer levels (12.6 µg/mL). Contrast-enhanced CT demonstrated poor opacification of the popliteal and distal arteries in the early arterial phase (Figure 2A), although delayed images showed restored enhancement. Additionally, delayed-phase images revealed stenosis of the left iliac vein (Figure 2B) and a filling defect extending from the iliac vein to the femoral vein, consistent with venous thrombosis (Figure 2C). Based on these findings, venous congestion due to DVT was considered, and iliac-type PCD was strongly suspected, with concerns for secondary limb ischemia accompanied by progressive worsening of paralysis; therefore, an emergency surgical intervention was indicated. Approximately five hours after the sudden exacerbation of symptoms, continuous intravenous unfractionated heparin infusion was started at 10,000 units/day; the patient underwent emergency angiography and thrombectomy.

**Figure 3:**
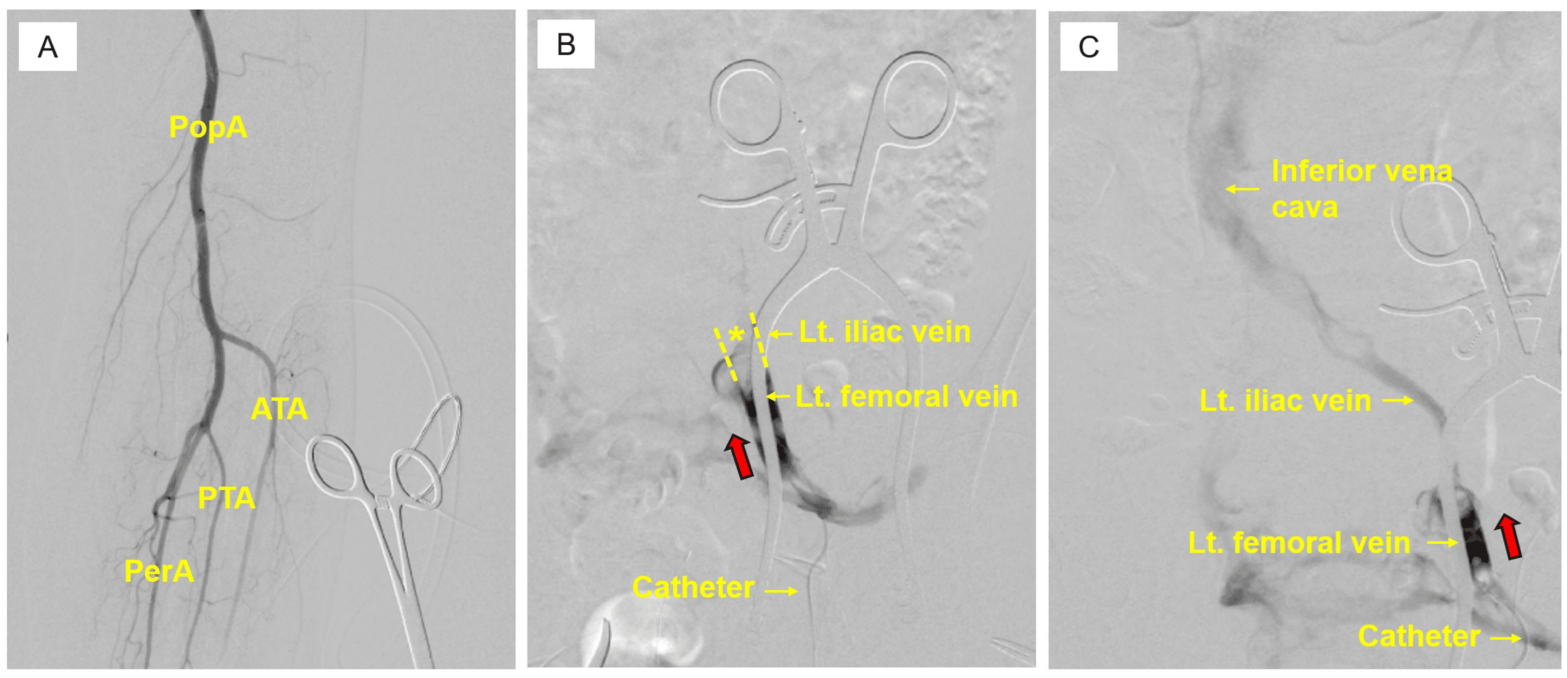
Intraoperative angiographic findings. (A) Angiography showing no filling defects or occlusive lesions in the infra-popliteal arteries. (B) Pre-thrombectomy imaging: ascending venography (red arrow) showing no opacification of the iliac vein or more proximal segments (*). (C) Post-thrombectomy imaging: contrast injection into the left femoral vein toward the proximal direction (red arrow) resulted in successful opacification of the iliac vein and inferior vena cava. Lt: left; PopA: popliteal artery; ATA: anterior tibial artery; PTA: posterior tibial artery; PerA: peroneal artery.

The patient was placed under general anesthesia. Then, a 5-cm incision was made in the left inguinal lesion, and the left common femoral artery and femoral vein were exposed. To assess ALI, we performed an initial angiography through the left common femoral artery. Given the presence of delayed opacification of the distal arteries but no evidence of arterial occlusion, ALI was excluded (Figure 3A). The femoral vein was markedly distended, and no thrombosis outflow was observed via venotomy. Contrast injected proximally from the femoral vein showed no opacification beyond the iliac vein (Figure 3B). After retrieving a substantial amount of dark-red thrombus by surgical thrombectomy using a 5-Fr Fogarty balloon catheter (Edwards Lifesciences, Irvine, CA, USA) and a Yankauer suction catheter (Medtronic, formerly Covidien, Minneapolis, MN, USA), proximal blood outflow was successfully restored. After thrombectomy, venography demonstrated smooth opacification up to the inferior vena cava (Figure 3C).

**Figure 4:**
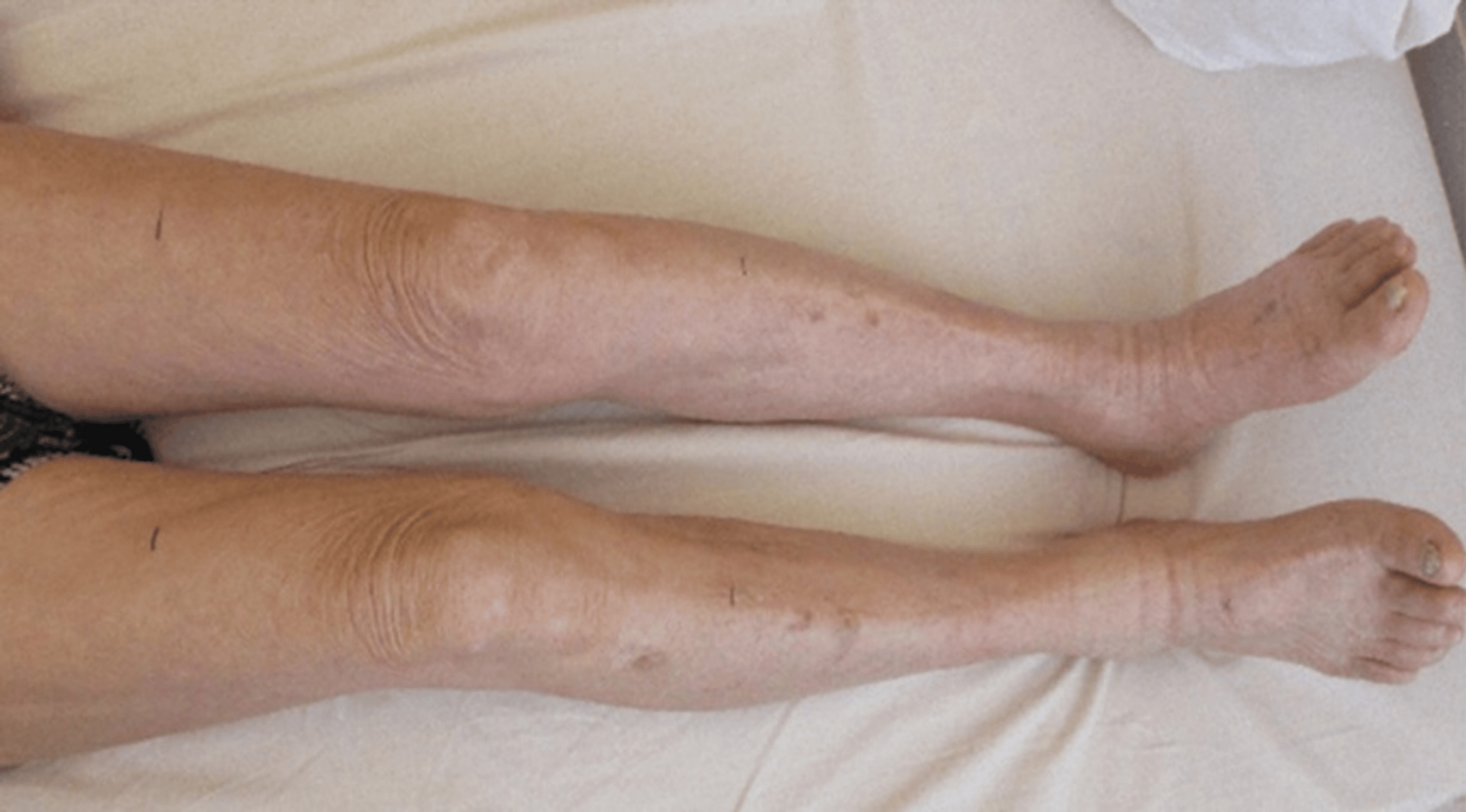
Improved pain, swelling, cyanosis, and paresis of the left lower limb.

**Figure 5:**
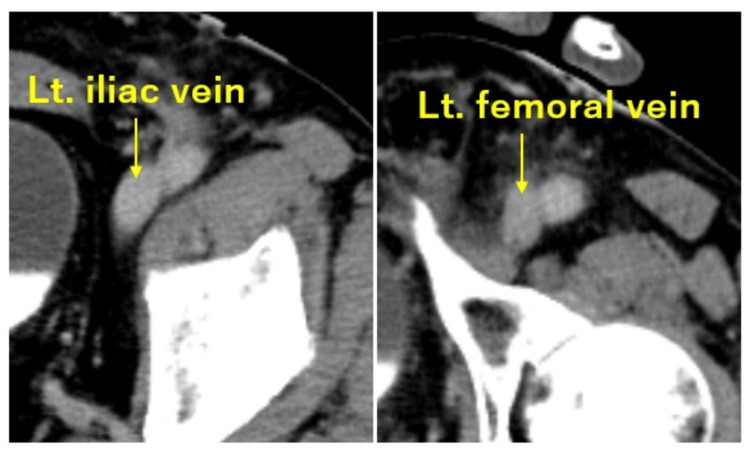
Postoperative computed tomography findings. No thrombus was detected in the iliac or femoral veins. Lt: left.

The elastic bandage was continued postoperatively. Limb pain, swelling, and cyanosis resolved (Figure 4), peripheral arterial pulses became palpable, and paresis improved. Continuous intravenous unfractionated heparin infusion was administered with a target activated partial thromboplastin time of 50-65 s, and a direct oral anticoagulant was initiated on postoperative day four. The postoperative course was uneventful. Postoperative CT confirmed the absence of venous thrombosis (Figure 5) and no evidence of malignancy associated with cancer-related hypercoagulability. Accordingly, he was discharged on postoperative day 14.

## Discussion

**Figure 6:**
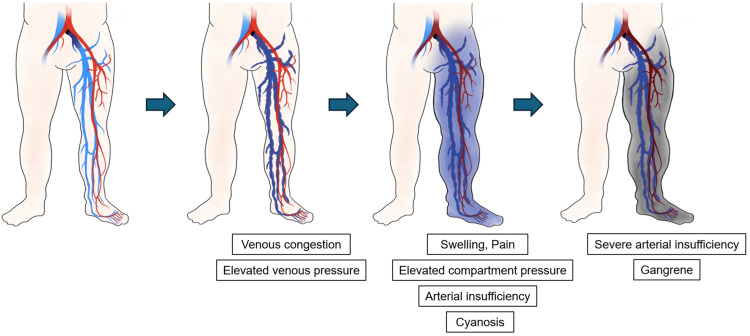
Clinical course of phlegmasia cerulea dolens. Initially, massive venous thrombosis causes venous congestion and increases venous pressure. Progressive edema results in swelling, severe pain, increased compartment pressure, and arterial insufficiency, leading to cyanosis. In the terminal stage, profound arterial compromise results in tissue necrosis and venous gangrene. All images are original and created by the authors.

PCD is a rare and severe manifestation of DVT, characterized by acute painful limb swelling and cyanosis caused by extensive venous thrombosis with markedly elevated venous pressure, which may progress to limb ischemia and life-threatening complications [1]. Prompt management of venous occlusion is crucial to prevent limb-threatening complications, given the high amputation (20%-50%) and mortality rates (25%-40%) reported [4]. Common risk factors for PCD include a history of venous thrombosis, older age, prolonged immobilization, malignant, surgery, trauma, pregnancy, use of oral contraceptives or hormonal therapy, and venous insufficiency [5]. Venous hypertension due to central venous thrombosis can cause painful limb swelling and cyanosis. As the condition progresses, elevated compartment pressure may impair arterial blood flow, resulting in limb ischemia, which can eventually lead to limb necrosis (Figure 6). In this case, despite the absence of conventional risk factors, the compression and stenosis of the left iliac vein between the common iliac artery and the lumbar vertebrae, as well as the cross-legged sitting position, may have contributed to the development of PCD.

The treatment strategy includes surgical thrombectomy combined with anticoagulation therapy, as timely and effective intervention is crucial for preserving limb viability. Grade III PCD, defined as venous gangrene in the PCD severity classification proposed by Chinsakchai et al. [4], is generally considered life-threatening with poor survival [6]. However, since PCD can clinically mimic ALI, prompt differentiation is essential. Severe edema and increased compartment pressures may mask peripheral pulse palpation [7]. Although ALI is often suspected in cases where early-phase contrast-enhanced CT shows reduced or absent arterial enhancement in patients with severe leg swelling and pain, PCD should also be considered. To clarify which is the case, delayed-phase imaging is crucial for evaluating venous patency and excluding deep venous obstruction.

Although a standardized treatment algorithm for PCD has not yet been established, various strategies--systemic heparinization, angioplasty, catheter-directed thrombolysis, surgical venous thrombectomy, and venous bypass (Palma procedure)--are used, each with advantages and limitations [4]. In cases with venous stenosis or compression, as in the present case, venous stent placement may be considered after thrombectomy; however, long-term patency remains uncertain [7]. Postphlebitic syndrome is a common complication of DVT, so using elastic compression stockings alongside anticoagulation is vital for long-term management [8]. In this case, an emergency balloon catheter thrombectomy was performed to rapidly restore venous return. Given the residual iliac vein stenosis and the associated risk of recurrent PCD, postoperative anticoagulant therapy with an unfractionated heparin was provided more intensively during the acute phase, followed by oral anticoagulant therapy.

## Conclusions

We report a rare case of PCD successfully treated with emergency surgical venous thrombectomy. Because PCD can closely mimic ALI, contrast-enhanced CT with delayed-phase imaging should be performed in patients presenting with lower limb ischemia. Early intervention is critical for limb salvage and survival. In patients with residual venous stenosis, careful postoperative anticoagulation and long-term follow-up are necessary to prevent recurrence.
